# Histopathological and immunohistochemical approaches for the diagnosis of Pasteurellosis in swine population of Punjab

**DOI:** 10.14202/vetworld.2016.989-995

**Published:** 2016-09-18

**Authors:** Payal Bhat, Nittin Dev Singh, Geeta Devi Leishangthem, Amninder Kaur, Vishal Mahajan, Harmanjit Singh Banga, Rajinder Singh Brar

**Affiliations:** 1 Department of Veterinary Pathology, College of Veterinary Science, Guru Angad Dev Veterinary and Animal Sciences University, Ludhiana -141 004, Punjab, India; 2 Animal Disease Research Centre, Guru Angad Dev Veterinary and Animal Sciences University, Ludhiana- 141 004, Punjab, India

**Keywords:** histopathology, immunohistochemical, Pasteurellosis, scoring

## Abstract

**Aim:**

Infectious porcine bronchopneumonia, caused by *Pasteurella multocida*, is a widespread disease of major economic significance. Thus, the aim of the present study was to diagnose swine Pasteurellosis using gross, histopathological, and immunopathological approaches in the swine population of Punjab and to compare the efficacy of immunohistochemical (IHC) techniques with conventional diagnostic techniques.

**Materials and Methods:**

A total of 71 adult swine lung samples showing gross pneumonic changes were collected along with the associated lymph nodes to carry out the study. The collected samples were then processed for histopathological and IHC studies.

**Results:**

Out of the total 71 lung samples, 26 samples were found to be suspected for Pasteurellosis as per the microscopic changes observed, and out of these 26 samples, 16 cases were confirmed to be positive for Pasteurellosis by IHC. Varied macroscopic changes noted in lungs were pneumonic patches with consolidation of many lobes, congestion, and focal hemorrhages. Main lesions associated with lymph nodes were its enlargement and hemorrhages. Histologically, the lung showed fibrinous and suppurative bronchopneumonia, multifocal suppuration, thickening of septa with fibrin combined with cellular infiltration and edema. The higher IHC expression of *P. multocida* was seen in the bronchial epithelium besides in alveolar and bronchial exudate. Moreover, on comparing the histopathological and IHC scores which were calculated on the basis of characteristic microscopic lesions and number of antigen positive cells, respectively, a significant positive correlation (r=0.4234) was found.

**Conclusion:**

It was concluded that swine population of Punjab is having *P. multocida* infection. The gross and histopathological lesions can be helpful in the preliminary diagnosis of Pasteurellosis but needs to be supplemented by other immunodiagnostic tests. Moreover, IHC technique proved to be a specific, reliable, precise, and rapid technique to supplement these conventional methods of diagnosis for Pasteurellosis.

## Introduction

Swine mortality is an important factor affecting the economic viability and profitability of swine industry. There are several bacterial, viral, parasitic, and managemental diseases of pigs that cause direct financial loss to farmers. Of several diseases, infectious pneumonia(s) has been shown to cause the greatest economic losses in pig houses [1], and it contributes 14% of the impact on the total disease expenses.

Pig rearing has traditionally been the main occupation of socially backward classes, especially in Northeastern states of India. It plays an important role for improvement of socioeconomic condition of poor farmers in India and also other developing countries. Moreover, due to rising per capita income, growing urbanization, and unfolding globalization, there is a rapid change in dietary habits of the people and the demand for pork has swiftly increased worldwide in the recent years. Lately, pig farming has gained popularity in Punjab with the introduction of new technologies for rearing and breeding of pigs along with subsidies being provided by central and state government schemes. The estimated profit due to pig farming topped other subsidiary occupations such as dairying, poultry, and sheep rearing; but due to the prevalence of many diseases in swines which go undiagnosed, the poor farmers also suffer huge economic losses. In the most recent decades, the population has declined to approximately 12 million head from a high of 14 million in 2003, as indicated by the 18^th^ Livestock Census of India [2]. Among all, respiratory diseases were found to be the fifth most important cause of mortality in crossbred pig in India [3].

Currently, infectious diseases of multifactorial etiology dominate in pigs. Among these, infectious porcine bronchopneumonia is a widespread disease of major economic significance [4]. Mainly, it is characterized by bronchopneumonia [5]. A frequent finding among the infectious agents incriminated with bronchopneumonia in pigs is the Gram-negative bacterium *Pasteurella multocida* [6]. *P. multocida* is also one of the causative agents of Porcine Respiratory Disease Complex (PRDC); however, it is rarely considered to be the primary agent but rather constitutes a part of the PRDC [6]. It is believed that *P. multocida* plays an important role by accentuating lung lesions, but little is known about the mechanism behind it [6]. Lesions associated with *P. multocida* are usually characterized by exudation into bronchi and bronchioles, but necrosis of lung tissue and sepsis may also be commonly observed [7].

Moreover, the status of Pasteurellosis is not known in the swine population of Punjab, and there are no available data regarding the pathology of Pasteurellosis in Punjab, and elimination of infectious agents can be successfully achieved only if the agent in question can be accurately identified. Therefore, the present study was conducted to know the presence of Pasteurellosis in swine population of Punjab, and an attempt was made for early diagnosis of Pasteurellosis in swines using gross and histopathological lesions and then authenticating the results by immunohistochemistry (IHC).

## Materials and Methods

### Ethical approval

The present study was conducted after the approval of the Research Committee and the Institutional Animal Ethics Committee.

### Collection and processing of samples

A total of 71 tissue samples of pneumonic lungs and associated lymph nodes (mediastinal and bronchial) were randomly collected from slaughterhouse/butcher shops and post-mortem hall of Guru Angad Dev Veterinary and Animal Sciences University (GADVASU), Ludhiana. The relevant tissue samples were fixed in 10% neutral buffered formalin for histopathology and IHC studies. The tissues were then processed and the 4 μ thick tissue sections were cut out of the paraffin-embedded tissue blocks and stained with hematoxylin and eosin staining as per the protocol of Bancroft and Gamble [8].

### IHC

For IHC studies, section(s) were taken on poly-L-lysine coated slides and then subjected to a clearing which was followed by rehydration. The antigen retrieval was carried out in citrate buffer using EZ-Retriever^®^ (Biogenex, USA). These slides were then washed in phosphate buffer saline for 20 min. Serum blocking was done using normal horse serum, and subsequently, non-specific binding and endogenous peroxidase blocking were followed by overnight incubation with polyclonal antibody for *P. multocida* that was standardized at the dilution of 1:2500. It was then followed by 20 min incubation with secondary antibody (Vector, impress reagent kit; peroxidase universal antimouse/rabbit Ig). Color was developed with substrate diaminobenzidine (vector) and counterstained with Gill’s hematoxylin (Merck, Germany) stain. Omission of primary antibodies was used for negative control. Further, IHC scoring was done on the basis of antigen positive cells and classified as mild, moderate, and severe.

### Statistical analysis

The SPSS software was employed to statistically ascertain the relationship between the scoring of histopathological changes in the lungs from affected pigs and the immunohistopathological scoring of the histopathologically positive samples using suitable statistical methods and formulae (mean, standard deviation, and Pearson’s correlation) to derive out the significant correlations. This immunohistopathological scoring was done using the scoring scale of 0-3, where five sites in each tissue section were observed under the microscope, and subsequently, the scoring was done by seeing the number of positive cells.

## Results and Discussion

A total of 71 adult swine lung samples along with associated lymph nodes were collected for the study. The lungs which showed gross pneumonic changes were only collected for the study.

### Gross

**Figure 1:**
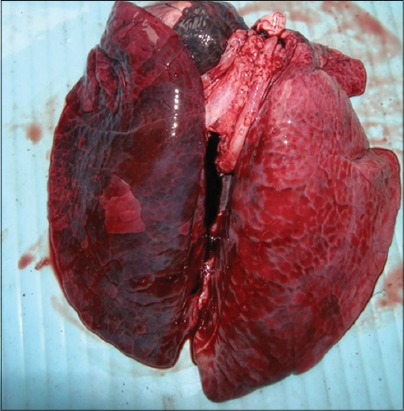
Lung grossly showing diffuse consolidation with hemorrhages in case of Pasteurella multocida.

**Figure 2:**
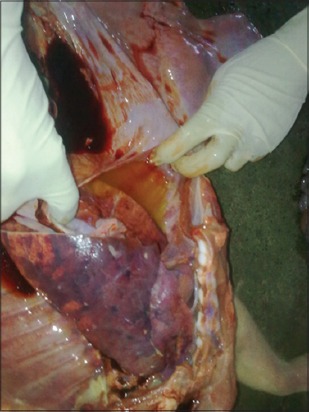
Diffuse lung consolidation with hydrothorax.

Main gross lung lesions observed in most of the cases were pneumonic patches in the lung with consolidation of many lobes, congestion, and focal hemorrhages (Figure-1), these observations were in accordance with Harish *et al*. [9] and Ghosh *et al*. [10]. Apart from it, patches of emphysematous areas were also noted and frothy exudate was seen oozing out after cutting the pneumonic patches of lungs. Beside it, small amount of frothy exudate in trachea and hydrothorax (Figure-2) was also noted as found by Tigga *et al*. [11] and Harish *et al*. [9]. Main lesions associated with lymph nodes were its enlargement and hemorrhages, and similar lesions were reported by Ghosh *et al*. [10].

### Histopathology

**Figure 3:**
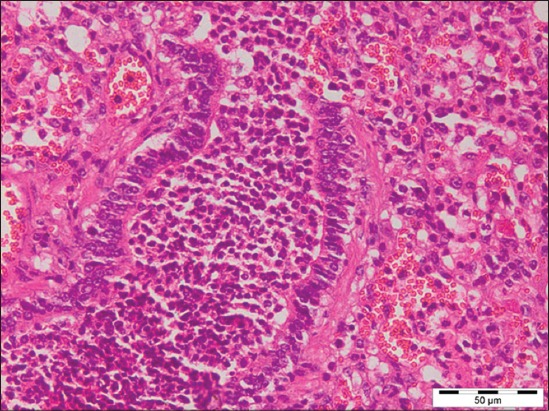
Severe suppurative bronchopneumonia in case of Pasteurellosis in pig (H and E, 400×).

**Figure 4:**
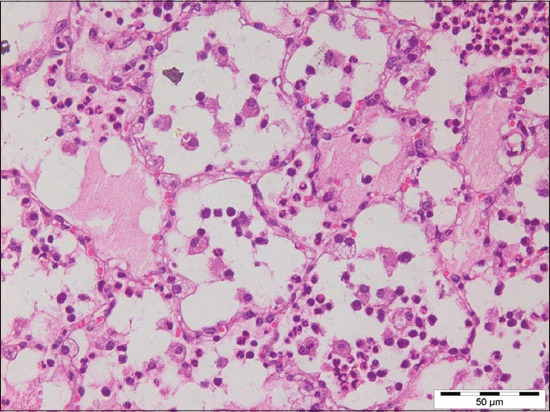
Thickening of alveolar septa with fibrin combined with cellular infiltration and edema in case of Pasteurellosis (H and E, 400×).

**Figure 5:**
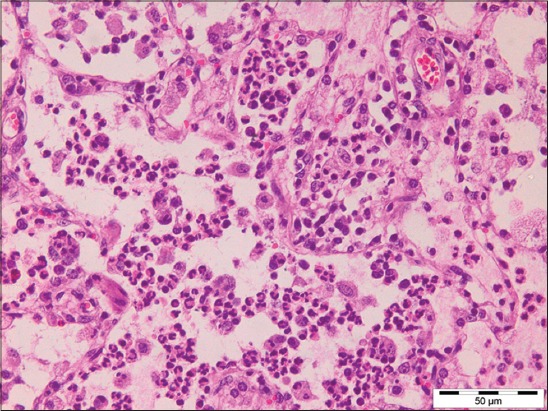
Alveoli filled with fibrinous exudate, erythrocytes, and polymorphonuclear cells in case of Pasteurellosis (H and E, 400×).

**Figure 6:**
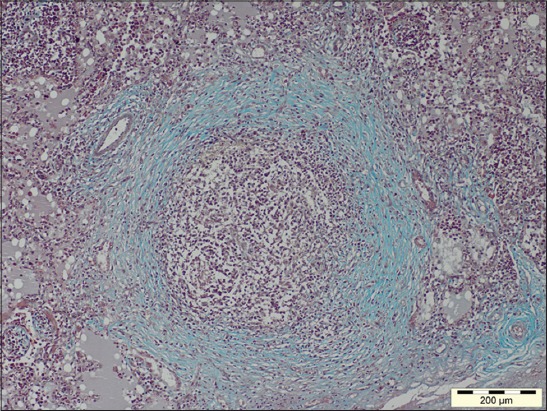
Focal area of necrosis surrounded by fibrous capsule in case of Pasteurellosis (Masson’s trichrome, 100×).

**Figure 7:**
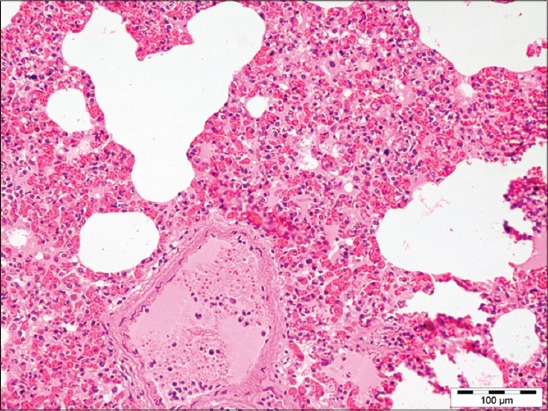
Diffuse hemorrhage in interstitial space of lung in case of Pasteurellosis (H and E, 200×).

**Figure 8:**
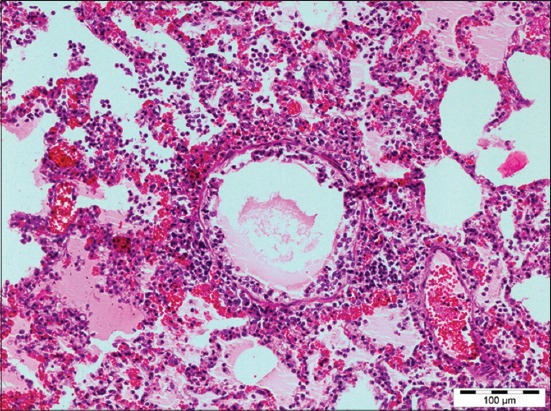
Bronchial epithelium degeneration, congestion, hemorrhage, and edema in lungs of pigs affected with Pasteurellosis (H and E, 200×).

**Figure 9:**
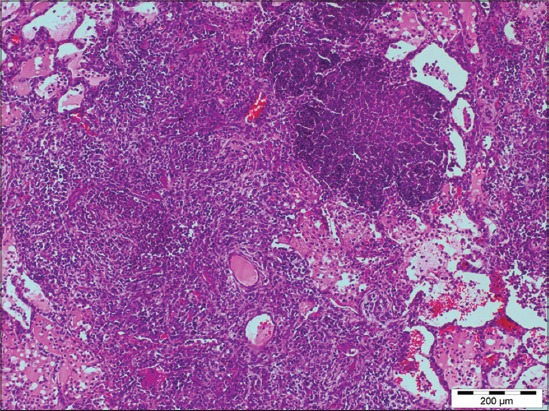
Multifocal necrotic areas in lung of pigs infected with *Pasteurella multocida* (H and E, 100×).

**Figure 10:**
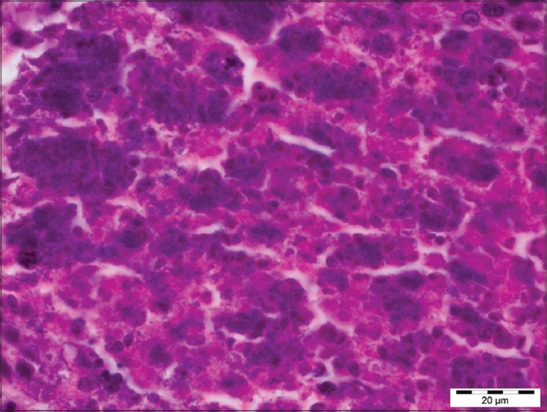
Necrotic areas showing the presence of bacterial colonies (H and E, 1000×).

**Figure 11:**
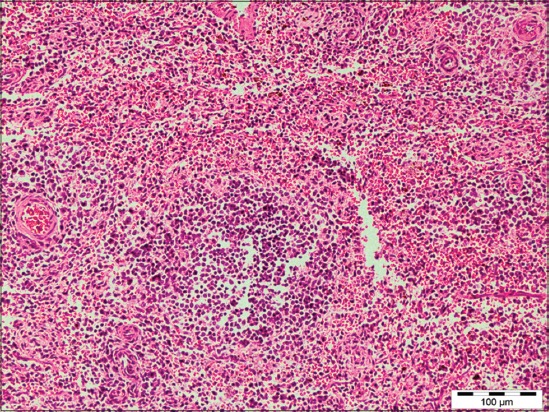
Lymph node showing diffuse hemorrhage and congestion in case of Pasteurellosis (H and E, 200×).

Out of 71 samples, 36.62% (26) cases were found to be showing characteristic histopathological lesions of *P. multocida* and were characterized by moderate fibrinous and suppurative bronchopneumonia (Figure-3), multifocal suppuration, thickening of septa with fibrin combined with cellular infiltration and edema (Figure-4). Alveoli were also infiltrated with fibrinous exudate, erythrocytes, and polymorphonuclear cells (Figure-5) with focal area of necrosis which was surrounded by a zone of fibrous capsule (Figure-6). In addition to it, thickening of pleura and subpleural hemorrhage(s) was also noted. Similar histological lesions in lungs were observed by Tigga *et al*. [11]. The lesions such as bronchioles filled with necrotic exudates containing inflammatory cells and multifocal hemorrhages in the interstitial spaces (Figure-7) were in accordance with Harish *et al*. [9]. In some cases, chronic active bronchopneumonia represented by exudation in the alveoli and bronchiole with different cell types, including neutrophils or a mixed cell population consisting of lymphocytes, neutrophils, and macrophages were also found. Similar histopathological lesions were reported by Ono *et al*. [12] and Pors *et al*. [13], along with it fibrous tissue proliferation was also present. Lesions observed in some cases were similar to that reported by Cardoso *et al*. [14], i.e. necrotizing bronchopneumonia associated with bronchial epithelium degeneration, hemorrhage, congestion, edema (Figure-8), fibrinous pleuritis, and emphysema in area adjacent to bronchopneumonia along with these necrotic areas with bacterial colonies were also seen (Figures-9 and 10). Main lesions associated with lymph nodes were found similar to that reported by Cardoso *et al*. [14] and Tigga *et al*. [11] that included hemorrhage, congestion, and lymphoid depletion (Figure-11).

According to an earlier study by Ciprian *et al*. [15], it was reported that *P. multocida* behaves as a secondary infectious agent along with main pathogens (*viz*., *Mycoplasma hyopneumoniae*, *Actinobacillus pleuropneumoniae*, and *Bordetella bronchiseptica*) causing pneumonia. *P. multocida* acts synergistically with other pathogens to increase respiratory lesions [6,16]. *P. multocida* is a normal commensal of upper respiratory tract of animals, and under any stress condition or infection with other agents, *P. multocida* proliferates and leads to occurrence of associated disease condition, thus with bronchopneumonia in pigs it is usually considered to be a secondary pathogen which is dependent on co-infection or immunosuppression of the host [17]. More neutrophil population in the present study is due to the fact that clearance of *P. multocida* from porcine lung depends on the presence of neutrophils as reported by Muller and Kohler [18]. Macrophage infiltration was also seen as neutrophils together with macrophages mediate phagocytosis and cytokine production [19].

The scoring of the various Pasteurellosis lesions in lung was done as shown in Table-1. As per the statistical analysis, it was observed that histopathological lesions associated with *P. multocida* showed mild degeneration and necrosis along with moderate changes such as inflammatory cell infiltration, hemorrhage, and bronchopneumonia.

**Table-1:** Histopathological scoring of lung lesions in case of Pasteurellosis.

Slide No.	Inflammatory cells infiltration and hemorrhage	Degeneration and necrosis	Bronchopneumonia
PB-1	2.8±0.4	1.4±0.49	2.8±0.4
PB-2	2.6±0.49	1.4±0.49	1.4±0.49
PB-3	1.4±0.49	2±0.63	1.2±0.4
PB-4	3±0	2.2±0.75	1.4±0.49
PB-5	1.6±0.49	1±0	1.4±0.49
PB-6	2.8±0.4	1.8±0.75	2.8±0.4
PB-8	2.4±0.8	1±0	2±0.63
PB-10	3±0	2.8±0.4	3±0
PB-18	2.2±0.75	0.2±0.4	0.2±0.4
PB-29	2.4±0.49	1.8±0.75	2.2±0.4
PB-34	1.2±0.4	0.2±0.4	0.2±0.4
PB-40	1.4±0.49	0.6±0.49	0.6±0.49
PB-50	1±0	0.4±0.49	0±0
PB-56	2.6±0.49	1.6±0.8	2.6±0.49
PB-60	0.6±0.49	0±0	0.2±0.4
PB-70	1.8±0.75	1±0	2.6±0.49
Mean lesion score	2.0±0.74	1.2±0.78	1.6±1.03

### Immunohistopathology

Around 26 cases that were suspected for Pasteurellosis by histopathological observations were further processed for detection of *P. multocida* antigen. Out of 26, a positive reaction was found in 61.54% (16/26) cases.

**Figure 12:**
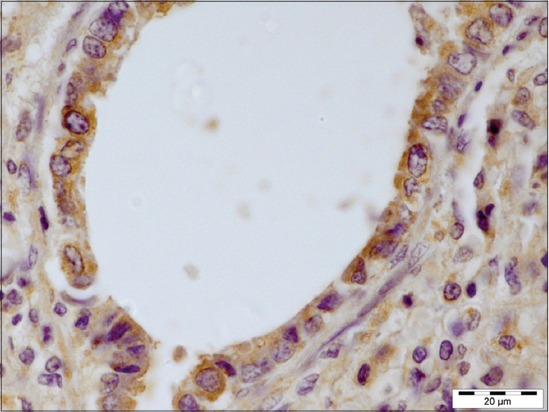
Presence of *Pasteurella multocida* antigen throughout the bronchial epithelium (immunohistochemical, 1000×).

**Figure 13:**
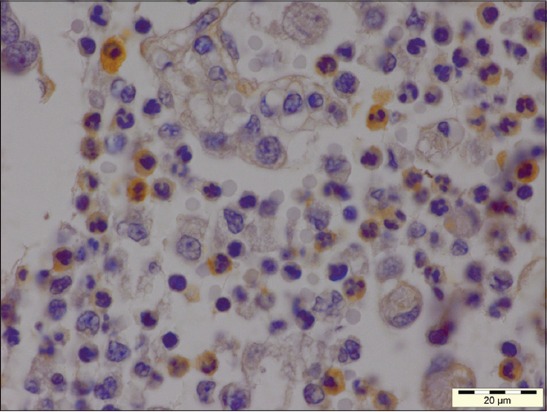
Immunolabeling *Pasteurella multocida* antigen in alveolar content mostly in neutrophils (immunohistochemical, 1000×).

**Figure 14:**
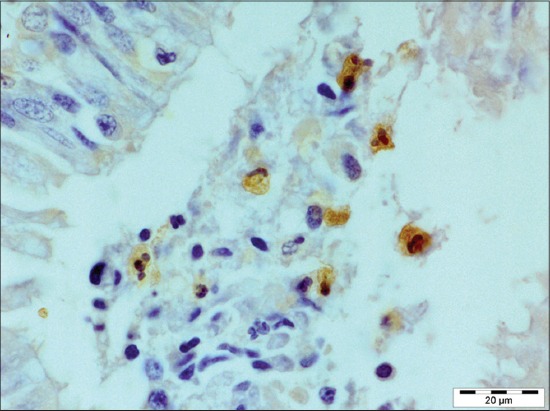
Immunolabeling for *Pasteurella multocida* antigen in bronchial content (immunohistochemical, 1000×).

**Figure 15:**
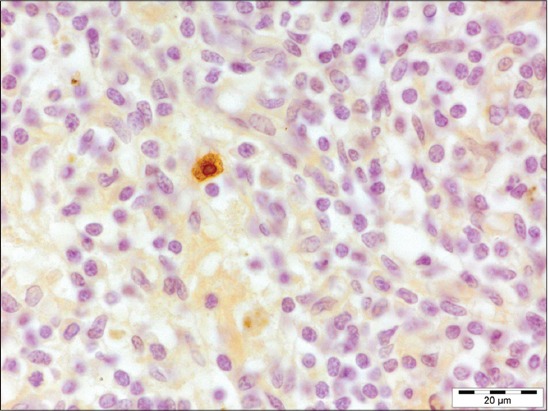
Mild positive reaction for *Pasteurella multocida* antigen seen in lymphocytes in lymph node (immunohistochemical, 1000×).

The affected lungs revealed antigen in bronchial epithelium (Figure-12), alveolar, and bronchial content having neutrophil and macrophages (Figures-13 and 14). These findings can be related to the findings of Ozyildiz *et al*. [20]. Mild positive reaction was also seen in lymphocytes in lymph nodes (Figure-15). The findings in the present study supported that the disease spread by the endobronchial way. In addition to it, the reaction in neutrophils was due to the fact that clearance of *P. multocida* from porcine lung depends on the presence of neutrophils. These findings were in accordance with the earlier study by Muller and Kohler [18].

### Statistical analysis

To statistically ascertain the relationship between the histopathological changes in the lung from affected pigs and the immunohistopathological scores, the immunohistopathological scoring of 16 out of 26 histopathologically positive samples was done using the scoring scale of 0-3.

**Figure 16:**
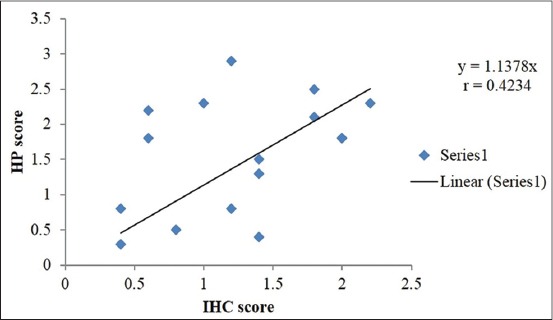
Correlation between histopathology (HP) score and immunohistochemistry (IHC) score in Pasteurellosis (Graph).

For a statistical comparison to be drawn, the mean and the standard deviation of the respective histopathological changes were also calculated as shown in Table-2. This mean represented the overall damage to the histoarchitecture, which was then compared with the IHC scoring of the respective cases using the SPSS software (SPSS for Windows version 16 INC, Chicago, Illinois). The correlation coefficient between the mean histopathological scoring and the IHC scoring was also found to be positive, r=0.4234 for the respective cases, which proves the reliability on IHC for the positive cases. Further, it can also be represented graphically, as depicted in Figure-16, where both of the curves follow a similar trend.

**Table-2:** Overall histopathological scoring and the IHC scoring of lung lesions in Pasteurellosis.

Case No.	Inflammatory cells infiltration and hemorrhage (mean±SD)	Degeneration and necrosis (mean±SD)	Bronchopneumonia (mean±SD)	Overall histopathological score	IHC score (mean±SD)
PB-1	2.8±0.4	1.4±0.49	2.8±0.4	2.33±0.66	2.2±0.75
PB-2	2.6±0.49	1.4±0.49	1.4±0.49	1.8±0.56	2±0
PB-3	1.4±0.49	2±0.63	1.2±0.4	1.53±0.33	1.4±0.8
PB-4	3±0	2.2±0.75	1.4±0.49	2.2±0.65	0.6±0.8
PB-5	1.6±0.49	1±0	1.4±0.49	1.33±0.25	1.4±0.8
PB-6	2.8±0.4	1.8±0.75	2.8±0.4	2.47±0.47	1.8±0.98
PB-8	2.4±0.8	1±0	2±0.63	1.8±0.59	2±0.63
PB-10	3±0	2.8±0.4	3±0	2.94±0.09	1.2±0.75
PB-18	2.2±0.75	0.2±0.4	0.2±0.4	0.87±0.94	1.2±0.75
PB-29	2.4±0.49	1.8±0.75	2.2±0.4	2.13±0.25	1.8±0.75
PB-34	1.2±0.4	0.2±0.4	0.2±0.4	0.53±0.47	0.8±0.4
PB-40	1.4±0.49	0.6±0.49	0.6±0.49	0.87±0.37	0.4±0.49
PB-50	1±0	0.4±0.49	0±0	0.47±0.41	1.4±1.02
PB-56	2.6±0.49	1.6±0.8	2.6±0.49	2.27±0.47	1±0.89
PB-60	0.6±0.49	0±0	0.2±0.4	0.27±0.25	0.4±0.49
PB-70	1.8±0.75	1±0	2.6±0.49	1.8±0.65	0.6±0.49

IHC: Immunohistochemical, SD: Standard deviation

## Conclusion

It was concluded that IHC staining is a precise, specific, rapid, and reliable method to demonstrate the Pasteurella antigen in the lung tissues of Pasteurella infected pig. Thus, IHC along with other conventional methods as gross and histopathological examination can be used for diagnosis of Pasteurellosis, i.e., an important respiratory disease of swine. Moreover, it has been reported for the first time that Pasteurellosis is one of the main respiratory diseases prevalent in swine population of Punjab.

## Authors’ Contributions

NDS and PB initiated research concept and design, collection, and analysis of data was compiled by PB, HSB, GDL, VM, and AK. The interpretation of HP was done by NDS and PB. Slide’s photography was done by PB and NDS. NDS, HSB, and RSB critically reviewed the article, whereas the final approval of the article was done by PB, NDS, HSB, and RSB. All authors read and approved the final manuscript.
